# Chloroform in Labor

**Published:** 1896-02

**Authors:** Ben. H. Brodnax

**Affiliations:** Brodnax, La.


### CHLOROFORM IN LABOR.

BY D. BEN. H. BRODNAX, BRODNAX, LA.

There has been much said relative to the action of this useful drug during labor, and so much has been written of its dangers as productive of or inducing post-partum hæmorrhage, that I have known some physicians who refuse to use it for fear of unpleasant sequelæ.

Nearly every medicine must be used with an eye to idiosyncrasy, and I am led to think much of the unpleasant effects recorded are to be laid to this peculiarity. It is well-known that chloroform, like opium, has an excitement period prior to its legitimate effect, during which, I have observed tremors of the body and limbs as great as in convulsions caused by nervous shock. In one case, the woman voided her urine in a full stream, as occurs in some hysterical cases. Also, there must be some care shown as to the patient, particularly when she has suffered from effects of recent malarial influence during gestation; as neither the blood nor veins of the body are of good quality and tone.

I believe there is no record of death during labor traceable to chloroform, the excitement of the system generally preventing too profound action of the drug. I have found in my practice thus far that it is safe to precede chloroform with chloral hydrate, it to be given in ten-drop doses, the chloroform to follow in a few minutes.

Let me suggest a plan which has for many years been a favorite of mine. I fix the cone or wet a handkerchief and let the woman hold it in her hand, to apply herself. It is most common when she is sufficiently under influence that she will let the hand drop from her face, to be replaced as soon as a new pain awakes her to her troubles. I do not think it best for the anæsthetic to be held by an attendant (often *we* have none), as in cases easily susceptible, there might be an unpleasant overdose.

In no case should the patient be unconcious of pain; sufficient to blunt sensibility is enough. At the same time, there will not be the nausea and headache which some have to suffer as an after effect.

I said *drops* of chloral. The amount of the drug in your vial is to be just covered with good glycerine, and you will have a clear saturated solution, one drop being about three-fourths of a grain of the drug.

The convenience of this is its easy and quick solution with the necessary amount of water, avoiding delay in dissolving the crystals. Another value of chloral is that, unlike morphine, it does not serve to intensify the constipated condition of the bowels attending the lying-in period. Sometimes, the first dose will nauseate, and vomiting may follow; but if the dose is repeated immediately, it will be retained.

Even this nausea is not a misfortune, as it aids the relaxation which is set up by the anæsthetic. I have noted two cases in which chloroform entirely stopped the action of the uterus, one in the practice of my friend, Dr. George H. Ogbourne, and one in my own. Strange to say, both were negroes. In my case, I found that another dose of chloral prevented this effect, by requiring less of the chloroform to produce ease.

In one case, recently, I observed there was a little more free hæmorrhage at the birth (exit) of the body of the child. But as it ceased immediately and the womb remained solidly contracted, I believe the chloroform had nothing to do with it. It was a lady who had been suffering for several weeks with chills and fever and was much reduced in strength and condition below usual standard.

If my brethren will try the plan set forth, I think they will be agreeably surprised at its simplicity and effectiveness. I do not know that there is anything new in it. The idea of the chloral was set forth in an article written by myself and read by Dr. Oscar H. Allis, before the Philadelphia County Society, in June, 1894. I had been using it that way for a number of years. Sometimes the amount of chloroform following was almost trifling to produce profound sleep and coma. Those curious to read the article, will find it in *Medical and Surgical Reporter*, July 7, 1894, pages 6 and 7.

One point more. In my practice, I find neither chloroform nor chloral seem to be incompatible with any of the coal tar derivatives (so-called) and acetanelid and lactophenene are very pleasant helps in time of need. Antikammia also is pleasant by itself and its combination with quinine is "simp'y splendid." If I am not mistaken, from its effects, the antikammia base is acetanelid.

Always, the main point is, in every way to give all the ease by every means that you can with safety in this the greatest glory and trial of woman.

---

"LOST MANHOOD."—Saw Palmetto is the "true vigor of life" in pre-senility. There is no country so prolific of sexual disorders and loss of virile power as Utah. A man cannot satisfy a dozen vigorous women and retain his full powers, and yet this, until recently, was what many were called upon to do. For this state of affairs, Saw Palmetto is *the* remedy, and I have had opportunity to put it to crucial test in very many stubborn cases—in pre-senility, prostatic troubles, ovarian pains, wasting testicles, wasting mammary glands, etc.—with universal success.

Early in May, 1895, there came to me a man of robust frame, aged fifty-one, who complained of difficulty in voiding urine and great tenderness in prostatic portion of urethra. About a year ago his urine began to pass with less force, and finally, with no force at all; he also noticed a weakening of virile power, and seldom had erections. His wife, a buxom blonde, several years his junior, had also noticed his inability to perform family duty as of yore; she would tantalize him and threaten to get a substitute, all of which nearly drove him to desperation. He became morose; everything went wrong; complained of feeling tired; could not endure any kind of exercise; mind was beclouded; sleep forsook him; penis was shrunken and cold—in fact, he was a badly played-out man. Fifteen drops fluid extract Saw Palmetto, taken four times daily, caused a more free flow of urine at the end of a week; he slept well, and felt better generally; also now complained of "drawing pains" in the spermatic cords. At the end of a fortnight his testicles, which had been much shrunken, proved to be gradually increasing in size; the urine flowed with more force, and the tenderness of the urethra had disappeared. He slept and rested perfectly, except when occasionally awakened by erections. At the end of a month he announced himself "once more a man;" said his wife no longer complained of a duty neglected, but declared his touch thrilled her with the same satisfaction and delight as in their early wedded life. His testicles were now larger than normal, but firm; and warmth and vigor had returned to such an extent he regrets the law that now prohibits a plurality of wives in Utah.—DR. A. L. DAVIDSON, in *Medical Gleaner*.

